# Pregnancy-Associated Thrombotic Thrombocytopenic Purpura: Diagnostic Pitfalls, Therapeutic Strategies, and Emerging Paradigms

**DOI:** 10.3390/biomedicines14020441

**Published:** 2026-02-15

**Authors:** Vinesh Kumar, Chandini Madeswaran, Venkata Sunkesula, Sirisha Kundrapu

**Affiliations:** 1 Department of Pathology, The MetroHealth System, Case Western Reserve University School of Medicine, Cleveland, OH 44109, USA; vkumar@metrohealth.org (V.K.); cmadeswaran@metrohealth.org (C.M.); 2 Department of Gastroenterology, The MetroHealth System, Case Western Reserve University School of Medicine, Cleveland, OH 44109, USA; vsunkesula@metrohealth.org

**Keywords:** pregnancy-associated thrombotic thrombocytopenic purpura, ADAMTS13 deficiency, thrombotic microangiopathy, immune-mediated thrombotic thrombocytopenic purpura, congenital thrombotic thrombocytopenic purpura

## Abstract

**Background**: Thrombotic thrombocytopenic purpura (TTP) is a rare but life-threatening thrombotic microangiopathy (TMA) caused by severe deficiency of the von Willebrand factor–cleaving protease ADAMTS13. Pregnancy is a recognized trigger for both immune-mediated and congenital TTP and is associated with increased maternal and fetal morbidity. Clinical overlap with other pregnancy-associated TMAs, including preeclampsia and Hemolysis, Elevated Liver enzymes, and Low Platelet count (HELLP) syndrome, often delays diagnosis. This review synthesizes current evidence on pathophysiology, diagnostic uncertainty, and gestation-specific management of pregnancy-associated TTP, highlighting differences between immune-mediated and congenital disease. **Methods**: This is a narrative review. We performed a targeted literature search of PubMed/MEDLINE (from inception to December 2025) to identify English-language publications. The study types included were case reports/series, observational studies, large database studies, randomized trials, reviews, and relevant guidelines addressing TMA in pregnancy, with emphasis on immune-mediated and congenital TTP. Search terms included “pregnancy”, “thrombotic thrombocytopenic purpura”, “hereditary TTP”, “acquired TTP”, “ADAMTS13,” “thrombotic microangiopathy,” “HELLP,” “postpartum”, and “complement-mediated TMA” alone or in combination. The search was supplemented by manual screening of reference lists and key guidelines. Articles were selected based on relevance to diagnosis and management of pregnancy-associated TTP. Conference abstracts and non-peer-reviewed sources were not routinely included and were considered only when peer-reviewed evidence was limited. **Results**: Pregnancy-associated TTP remains a major diagnostic challenge due to overlapping clinical and laboratory features with other obstetric thrombotic microangiopathies. Distinguishing immune-mediated from congenital TTP is essential, as management and prognosis differ substantially. Prompt recognition and early initiation of therapeutic plasma exchange, immunosuppression, or prophylactic plasma therapy markedly improve maternal outcomes. Rapid ADAMTS13 testing, structured risk stratification, and multidisciplinary care are central to optimal management. Fetal outcomes are closely linked to gestational age at onset and timeliness of therapy. **Conclusions**: Early differentiation of TTP from other pregnancy-associated TMAs is critical for maternal and fetal survival. Advances in rapid ADAMTS13 diagnostics and emerging targeted therapies, including caplacizumab and recombinant ADAMTS13, offer opportunities to improve precision management and outcomes in future pregnancies.

## 1. Introduction

Thrombotic thrombocytopenic purpura (TTP) is one of the classic Thrombotic microangiopathies (TMAs) caused by a severe deficiency of the von Willebrand factor (vWF)–cleaving protease ADAMTS13 (A Disintegrin And Metalloprotease with Thrombospondin type 1 motif, member 13), typically defined as activity <10% of normal. This deficiency leads to accumulation of ultra-large vWF multimers, unchecked platelet aggregation, and widespread microvascular thrombosis. Severe ADAMTS13 deficiency may result from autoantibody-mediated inhibition (acquired or immune-mediated TTP (iTTP)) or from biallelic pathogenic variants in the *ADAMTS13* gene (hereditary or congenital TTP (cTTP) or Upshaw–Schulman syndrome) [1,2].

Pregnancy is a known trigger for both iTTP and cTTP, and while classic TTP remains rare in pregnancy compared with other TMAs, its frequency among pregnant women is substantially higher than in the general population (estimated ~1 in 25,000–100,000 pregnancies) [3]. During normal pregnancy, ADAMTS13 activity physiologically declines and vWF levels increase, contributing to a shifted ADAMTS13–vWF balance that may precipitate TTP in susceptible individuals. Reports suggest that pregnancy-associated changes in hemostasis can unmask both inherited and acquired ADAMTS13 deficiency, increasing maternal and fetal morbidity if not recognized promptly [4,5]. In addition, pregnancy-related immune modulation and the postpartum immune rebound may contribute to an increased susceptibility to iTTP, with a well-recognized clustering of disease onset and relapses in the late pregnancy and postpartum periods [6,7].

The incidence of TTP in pregnancy and the postpartum period (approximately 1 in 25,000–100,000 pregnancies) is markedly higher than the annual incidence in the general population (~1–6 per million) and overlaps clinically with other pregnancy-associated TMAs—such as preeclampsia; Hemolysis, Elevated Liver enzymes, and Low Platelets (HELLP) syndrome; and atypical hemolytic uremic syndrome (aHUS)—making diagnosis challenging [3]. Distinguishing hereditary from acquired TTP is crucial, as management strategies differ; hereditary TTP may present for the first time in pregnancy and often requires ongoing prophylactic plasma therapy [8,9].

Advances in rapid ADAMTS13 activity testing, prompt therapeutic plasma exchange (TPE), and adjunct immunosuppressive therapies have markedly improved maternal survival. Moreover, prophylactic plasma infusions in cTTP and emerging agents such as caplacizumab and recombinant ADAMTS13 provide promising preventive and therapeutic options. Early recognition and multidisciplinary management remain essential for optimizing both maternal and fetal outcomes [10].

Despite multiple narrative reviews, pregnancy-associated TTP remains underrecognized as a distinct clinical entity with unique diagnostic pitfalls, management constraints, and long-term implications. Recent advances in rapid ADAMTS13 testing, targeted therapies, and biomarker-guided relapse prevention warrant an updated, pregnancy-focused synthesis integrating hematologic and obstetric perspectives.

## 2. Literature Search

We performed a targeted literature search of PubMed/MEDLINE from inception through December 2025 using the terms “pregnancy”, “thrombotic thrombocytopenic purpura”, “hereditary TTP”, “acquired TTP”, “ADAMTS13,” “thrombotic microangiopathy,” “HELLP,” “postpartum”, and “complement-mediated TMA” alone or in combination. The search was supplemented by manual review of reference lists from key articles and relevant society or working-group guidelines to identify additional publications. We included English-language case reports and case series, observational studies, large database studies, randomized trials, narrative and systematic reviews, and clinical practice guidelines focused on pregnancy-associated iTTP or cTTP. Articles were selected based on clinical relevance to diagnosis, differential diagnosis, and management in pregnancy and the postpartum period, with preference given to more recent evidence when available. Conference abstracts and non-peer-reviewed sources were not routinely included and were considered selectively only when peer-reviewed evidence was limited.

## 3. Epidemiology and Risk Factors

### 3.1. Incidence of iTTP and cTTP in Pregnancy

Large observational cohorts and registry-based studies have demonstrated substantial geographic and clinical variation in the incidence and outcomes of thrombotic thrombocytopenic purpura (TTP). The reported incidence of congenital TTP (cTTP) is rare, estimated at approximately 0.2–0.4 cases per million person-years, whereas the incidence of immune-mediated (idiopathic) TTP (iTTP) ranges from ~1.8 to 4 cases per million person-years in the general population. Overall, the annual incidence of all forms of TTP is estimated at 2–11 cases per million persons [11].

Severe ADAMTS13 deficiency during pregnancy has been reported in approximately 1 in 17,000 to 1 in 200,000 pregnancies, reflecting both cTTP and iTTP. Approximately 50% of acute TTP presentations occur in women of childbearing age, and pregnancy-associated TTP accounts for roughly 12–25% of adult-onset cases, underscoring pregnancy as a high-risk period for both first presentation and relapse [1,8].

### 3.2. Risk Factors, Gestational Timing and Recurrence

Pregnancy, including the postpartum period, and infection are among the most frequently reported triggers of acute episodes of both cTTP and iTTP. A comprehensive systematic review of observational studies showed that common acute triggers are pregnancy and infection, and rates of iTTP exacerbation varied widely, from approximately 2.4% to 63.1%, reflecting differences in study design and exacerbation definitions across cohorts [12].

Large registry datasets indicate that cTTP comprises a higher proportion of pregnancy-onset TTP than would be expected from its frequency in the general adult TTP population. In the French TMA Registry, ∼27% of pregnancy-onset TTP cases were cTTP, and UK TTP Registry data similarly show an overrepresentation of hereditary disease among pregnancy-associated presentations [8,11].

Despite the increased relative frequency of TTP during pregnancy compared with non-pregnant states, the absolute incidence of pregnancy-associated TTP remains low relative to other pregnancy-associated TMAs such as preeclampsia and HELLP syndrome [13]. TTP can occur at any gestational age but is most recognized in the third trimester and during the postpartum period, consistent with clinical series describing temporal clustering of acute episodes in these phases [11,14].

Contemporary diagnostic approaches emphasize that TTP should be suspected in patients presenting with MAHA and thrombocytopenia even in the absence of classic pentad (fever, neurological abnormalities and renal dysfunction). As a result, prior studies and registries that relied on classic diagnostic criteria may underestimate the current incidence of acute TTP, because “non-pentad” presentations would be less likely to be recognized [12].

## 4. Pathophysiology

In 1924, Eli Moschcowitz first described a case of fatal illness consistent with TTP in a 16-year-old girl presenting with fever, hemolytic anemia, neurologic deficits, and coma. It was postulated that the disease was caused by platelet-rich thrombi within microcirculation [15]. In 1955, Gasser et al. reported a series of five pediatric cases of hemolytic uremic syndrome (HUS), defining its classic triad of thrombocytopenia, non-immune MAHA, and acute kidney failure [16]. Subsequently, in 1960, Schulman et al. described a child with relapsing thrombocytopenia responsive to plasma infusion, suggesting a deficiency of a circulating plasma factor required for normal platelet regulation [17]. Upshaw later reported similar cases, and this condition is now recognized as Upshaw–Schulman syndrome, which is the congenital form of TTP [18].

These early astute clinical observations that anticipated the underlying pathophysiology were confirmed in the 1980s and 1990s with the technological advances and the availability of diagnostic tools. In 1982, Moake et al. identified ultra-large VWF multimers in patients with TTP, implicating these multimers in microvascular platelet aggregation [19]. In 1985, Karmali et al. established the association between HUS and infection with Shiga toxin-producing Escherichia coli [20]. In 1996, Furlan et al. and Tsai HM independently isolated a plasma protease capable of cleaving ultra-large VWF multimers [21,22]. By 2001, multiple groups identified this protease as ADAMTS13, a zinc-containing metalloprotease essential for regulating VWF multimer [23].

**Figure 1:**
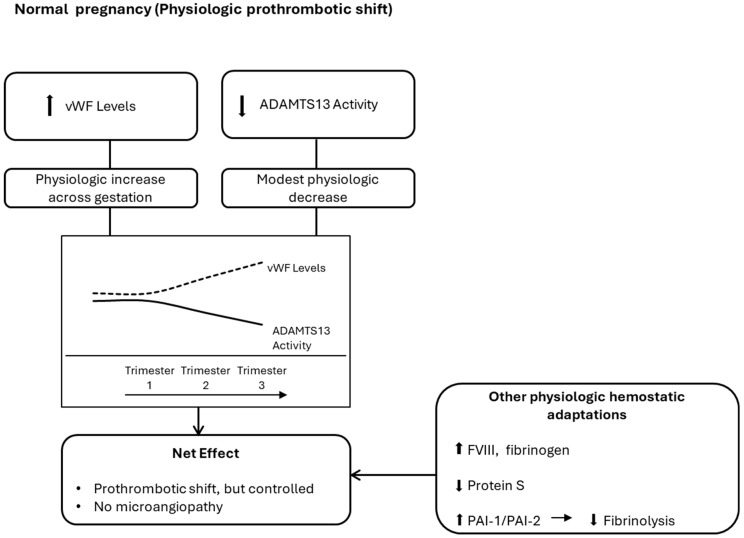
ADAMTS13–vWF axis in normal pregnancy: Physiologic adaptation. The upward arrow means “Increased” and the downward arrow means “Decreased”.

Pregnancy is associated with increased von Willebrand factor (vWF) levels and a physiologic decline in ADAMTS13 activity, resulting in an altered vWF–ADAMTS13 balance. In susceptible individuals, further reduction in ADAMTS13 activity due to autoantibody-mediated inhibition or genetic mutations leads to accumulation of ultra-large vWF multimers, platelet-rich microthrombi, and microangiopathic hemolysis. Pregnancy-related endothelial activation may further amplify this process (Figure 1) [6,24].

During healthy pregnancy, vWF levels rise and ADAMTS13 activity may decrease modestly across gestation, creating a physiologic prothrombotic shift that remains regulated without microvascular thrombosis. This shift occurs alongside broader hemostatic adaptations, including increased procoagulant factors (e.g., factor VIII and fibrinogen), reduced protein S activity, and increased placental/endothelial plasminogen activator inhibitors (PAI-1 and PAI-2) with relative suppression of fibrinolysis, collectively supporting hemostasis at delivery [6,24].

Emerging single-cell studies highlight platelet heterogeneity in inflammatory and thrombotic diseases. Distinct platelet subpopulations linked to coagulation activation, hypoxic/stress transcriptional programs, and clinical severity have been identified in conditions such as COVID-19, sepsis, and systemic lupus erythematosus. Whether similar platelet subpopulations exist in TTP, particularly during pregnancy, and which subsets most strongly drive vWF-mediated platelet adhesion and microvascular thrombosis remain important open questions. Future studies integrating platelet single-cell profiling with ADAMTS13 activity, vWF multimer patterns, and clinical outcomes may clarify heterogeneity in thrombotic risk and treatment response [25].

### 4.1. Congenital TTP

**Figure 2:**
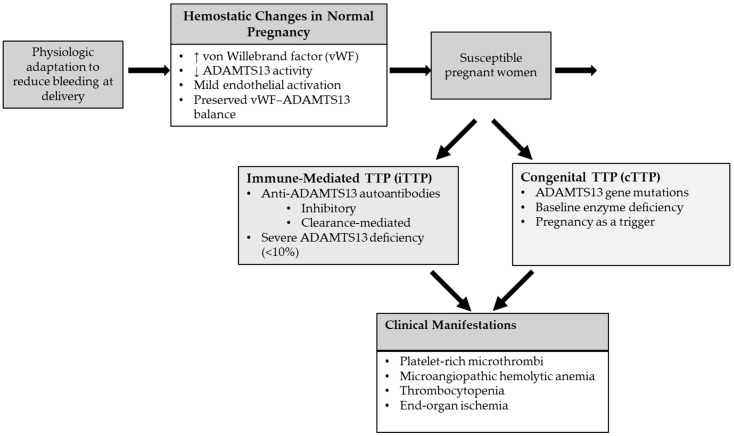
Pathophysiology of thrombotic thrombocytopenic purpura in pregnancy. The upward arrow means “Increased” and the downward arrow means “Decreased”.

Congenital TTP (cTTP) is an autosomal recessive disorder caused by biallelic germline pathogenic variants in the ADAMTS13 gene, resulting in severe lifelong deficiency of ADAMTS13 activity, which is often undetectable or <10% of normal levels (Figure 2) [26,27]. Numerous pathogenic ADAMTS13 variants have been described, including missense, nonsense, frameshift, splice-site mutations, and insertions or deletions [28,29,30]. Although most pathogenic ADAMTS13 variants are unique to individual families, a small number of mutations recur across multiple unrelated families.

Data from the Hereditary TTP Registry, which aggregates genetic and clinical information from patients with cTTP and their relatives across multiple countries, have demonstrated extensive genetic diversity and marked clinical heterogeneity in this disorder. Substantial variability has been observed in age at presentation, frequency of relapses, organ involvement, and disease severity, even among individuals sharing identical genotypes, including siblings [31]. These findings indicate that disease expression is not determined by genotype alone, but rather is influenced by additional modifiers such as pregnancy, infections, environmental factors, and other genetic variations. Consistent with this, recent largescale genomic studies have identified an expanding number of additional ADAMTS13 variants predicted to be pathogenic, further highlighting the remarkable genetic diversity underlying cTTP [28,29].

Heterozygous carriers of a single ADAMTS13 pathogenic variant retain sufficient residual enzyme activity to prevent ultra-large vWF-mediated microvascular thrombosis and typically do not develop clinical TTP [30]. However, these individuals have lower baseline ADAMTS13 activity compared with the general population and may have an increased long-term risk of cardiovascular or cerebrovascular disease, suggesting a broader role for ADAMTS13 in thrombotic risk beyond overt TTP [28,31,32].

### 4.2. Immune-Mediated TTP

Overall, the most common cause of severe ADAMTS13 deficiency is the development of autoantibodies against ADAMTS13. In most cases of iTTP, these autoantibodies are inhibitory, directly blocking the proteolytic activity of ADAMTS13. In a smaller subset of patients, non-inhibitory (non-neutralizing) autoantibodies predominate; these antibodies do not directly inhibit enzymatic function but instead accelerate the clearance of ADAMTS13 from the circulation, leading to severe functional deficiency [33,34].

Although no specific environmental or genetic risk factors have been definitively established for the development of anti-ADAMTS13 autoantibodies, epidemiologic studies have consistently demonstrated that iTTP occurs more frequently in young women and in individuals of Black race. In addition, an increased incidence of iTTP has been observed in patients with coexisting autoimmune diseases, particularly systemic lupus erythematosus (SLE), supporting a shared autoimmune predisposition (Figure 2) [35,36,37].

### 4.3. Pregnancy-Specific Factors

Normal pregnancy is associated with physiological changes in hemostasis, including a progressive decrease in ADAMTS13 activity, with the most pronounced reduction occurring during the third trimester, and corresponding increase in VWF levels. This leads to altered vWF–ADAMTS13 balance, which is a physiologic adaptation to reduce bleeding at delivery. Despite these changes, ADAMTS13 activity in healthy pregnancy typically remains above the critical threshold required to prevent the development of TTP. However, this altered balance may precipitate TTP in susceptible individuals (Figure 2) [38,39].

Preeclampsia is a pregnancy-specific hypertensive disorder characterized by systemic endothelial dysfunction and activation. It results in increased vWF release, platelet consumption and microangiopathic hemolysis, which overlap with both TTP and HELLP syndrome, making clinical distinction challenging in pregnancy-associated TMA. Severe ADAMTS13 deficiency strongly supports a diagnosis of TTP, and it is critical to test ADAMTS13 activity when a pregnant patient presents with an unexplained TMA [39]. Moreover, when endothelial activation occurs as in preeclampsia, vWF levels rise and ADAMTS13 activity falls, leading to a high VWF/ADAMTS13 ratio. Therefore, a high vWF/ADAMTS13 ratio reflects the severity of endothelial injury and has been shown to correlate with worse outcomes in several other conditions like COVID-19 infection, stroke, and sepsis [40].

Normal pregnancy is also associated with mild activation of the immune system to support placental development and protect against infections. This includes physiologic activation of the complement system, reflected by increased circulating levels of C3 and C4 as well as activation fragments C3a and C5a [41,42]. This low-grade activation is counterbalanced by a concomitant increase in complement regulatory proteins, particularly factor H, which limits progression to terminal complement pathway activation. As a result, levels of the terminal complement complex soluble C5b-9 remain stable, indicating that complement activity during healthy pregnancy exists in a rebalanced state of immune homeostasis rather than overt complement-mediated injury [42,43]. Consequently, most pregnancies do not develop complement-mediated thrombotic microangiopathies. However, disruption of this balance due to genetic predisposition, infection, or preeclampsia may precipitate thrombotic microangiopathy [6,44].

## 5. Clinical Presentation

### 5.1. Maternal Features

In pregnancy, TTP often presents as a systemic thrombotic microangiopathy with rapid progression to multi-organ dysfunction, including neurologic involvement, acute kidney injury, cardiac ischemia, hepatic injury, and placental insufficiency. It typically presents with the classic triad of microangiopathic hemolytic anemia (MAHA), severe thrombocytopenia, and neurologic manifestations, including confusion, headache, seizures, or stroke, like TTP occurring outside of pregnancy. In some cases, additional features such as renal dysfunction and fever may be present, completing the traditional pentad. However, accumulating evidence demonstrates that the full pentad is uncommon at presentation, and current diagnostic criteria do not require its presence for initiation of treatment in suspected TTP, given the urgency of therapy and high associated mortality if treatment is delayed. It must be noted that it is impossible to differentiate between cTTP and iTTP based on the symptoms alone [11].

The threshold for suspicion of TTP during the early postpartum period should be low. Physiological changes associated with pregnancy and delivery including hormonal fluctuations, a hypercoagulable state, and immune modulation may act as triggers for the severe endothelial dysfunction characteristic of TTP. This period poses a unique diagnostic challenge, as the clinical manifestations of TTP often overlap with those of other obstetric complications [14,45].

The combination of the following key laboratory findings strongly suggests TTP: the presence of schistocytes on the peripheral blood smear, refractory thrombocytopenia, and elevated lactate dehydrogenase (LDH). Atypical presentations of TTP, though uncommon, can further complicate diagnosis. Reported atypical manifestations include acute pancreatitis (likely secondary to ischemia) and bloody diarrhea due to bowel ischemia, which may be misinterpreted as Shiga toxin-associated hemolytic uremic syndrome. In some cases, acute neurologic Manifestations, such as stroke or seizures, may precede the development of the classic laboratory features of MAHA and thrombocytopenia. In the setting of such atypical presentations, serial examination of the peripheral blood smear for schistocytes and ADAMTS13 activity testing are critical to establishing a timely diagnosis [14,46].

### 5.2. Fetal Outcomes

Studies have shown that TTP presenting during pregnancy is associated with worse fetal outcomes compared with uncomplicated pregnancies. Reported complications include miscarriage, preterm delivery, intrauterine growth retardation, intrauterine fetal death, stillbirth and neonatal death [47]. Adverse fetal outcomes are more frequent when TTP presents earlier in gestation, particularly during the first and second trimesters [8].

In a cohort of women with iTTP, up to 50% of pregnancies complicated by TTP relapse during gestation resulted in fetal death or miscarriage, and among pregnancies progressing beyond 20 weeks’ gestation, approximately 50% were complicated by preeclampsia or HELLP syndrome. Similarly, a case series of TTP reported fetal survival rates ranging from 58–65% when postpartum cases are excluded [11,48].

Because iTTP is often unpredictable, it may be associated with higher risk of adverse fetal outcomes compared with cTTP, in which outcomes may improve with early prophylactic plasma infusion in known cases. In pregnant women with known history of iTTP, evaluation of ADAMTS13 activity during pregnancy may help predict the risk of relapse and associated maternal and fetal complications [49,50]. Overall, fetal outcomes in pregnancy-associated TTP are closely linked to gestational age at presentation, disease severity, and timely intervention to restore ADAMTS13 activity.

## 6. Diagnostic Pitfalls in Pregnancy-Associated Thrombotic Microangiopathy

### 6.1. Complement-Mediated TMA

Previously known as atypical hemolytic uremia syndrome (aHUS), CM-TMA is a non-diarrheal hemolytic uremic syndrome that can affect both children and adults including pregnant women. CM-TMA has traditionally been viewed as a disorder of the alternative complement pathway, as many affected patients harbor pathogenic variants in genes encoding complement regulatory proteins. Dysregulation or uncontrolled complement activation leads to inflammation, platelet activation, endothelial injury, and activation of the coagulation cascade, culminating in microvascular thrombosis [43,44]. Renal endothelial cells are particularly sensitive to complement-mediated injury, which likely explains the predominance of severe acute kidney injury in CM-TMA [51].

However, up to 50% of patients lack identifiable complement gene variants, and only a minority of variant carriers develop overt disease, indicating that these variants function as predisposing rather than causative factors [52]. Among complement-amplifying triggers, pregnancy and infections are most frequently implicated (6,44). Accordingly, CM-TMA is best understood as a two-hit disorder, in which underlying susceptibility to complement dysregulation is unmasked by a triggering event, resulting in uncontrolled complement activation and TMA.

It is often challenging to clinically differentiate CM-TMA from TTP in pregnancy due to significant overlap in presenting features. However, accurate distinction is critical, as prompt initiation of appropriate therapy is essential and the management of these conditions is fundamentally different. TTP often features more prominent neurologic involvement with comparatively less severe renal injury, whereas CM-TMA more commonly presents with severe acute kidney injury, frequently requiring dialysis [51].

Laboratory findings further aid in differentiation. TTP is defined by severe ADAMTS13 deficiency (<10%), often accompanied by profound thrombocytopenia, while CM-TMA is associated with preserved or only mildly reduced ADAMTS13 activity and moderate thrombocytopenia [44,51,53]. From a temporal standpoint, TTP may occur during any trimester but most commonly presents in the second or third trimester, whereas CM-TMA classically manifests in the postpartum period, often triggered by delivery-related complement activation [11,44,54].

Overall, ADAMTS13 activity remains the key diagnostic discriminator guiding timely therapeutic decisions, with urgent plasma exchange required for TTP and complement inhibition indicated for CM-TMA (Table 1) [6,44,53].

### 6.2. HELLP Syndrome and Severe Preeclampsia

Differentiating TTP from other pregnancy-specific TMAs, particularly preeclampsia and HELLP syndrome, is challenging because of the overlapping clinical features (thrombocytopenia, MAHA and end-organ dysfunction). The relationship between TTP and HELLP syndrome in pregnancy is complex and may involve two distinct clinical scenarios: (a) The concurrent development of both TTP and HELLP syndrome and (b) TTP-driven placental dysfunction, in which a patient with pregestational TTP develops superimposed preeclampsia [55].

In cTTP, severe deficiency of ADAMTS13 activity (<10%) leads to widespread microvascular platelet-rich thrombosis, including within the placental circulation. This placental microthrombosis results in maternal vascular malperfusion, a key pathologic feature also observed in early-onset preeclampsia and HELLP syndrome. This shared placental pathology explains why women with cTTP may present with a HELLP-like phenotype, particularly early in pregnancy. However, placental ischemia in cTTP is directly driven by ADAMTS13 deficiency unlike the true HELLP syndrome where placental dysfunction is secondary to abnormal placentation and endothelial activation. Clinical improvement with fresh frozen plasma infusions supports TTP as the primary disease process rather than HELLP syndrome in such cases (Table 1) [56].

TTP should be strongly suspected when TMA presents 20 weeks before gestation or when severe neurologic manifestations or cardiac injury are present. Persistence or progression of thrombocytopenia and hemolysis after delivery should prompt evaluation for TTP, particularly with neurologic involvement or severe ADAMTS13 deficiency. In such cases, immediate measurement of ADAMTS13 activity should be performed, as severe deficiency (<10%) is highly specific for TTP. Prompt diagnosis and timely initiation of therapy are essential to optimize both maternal and fetal outcomes [6,11,53].

### 6.3. Acute Fatty Liver of Pregnancy (AFLP)

AFLP is a disorder caused by defects in fetal mitochondrial fatty acid β-oxidation, most commonly long-chain 3-hydroxyacyl-CoA dehydrogenase (LCHAD) deficiency. In this condition, the mother, a heterozygous carrier, is exposed to toxic long-chain 3-hydroxyacyl fatty acid metabolites derived from the fetus and placenta, leading to micro-vesicular hepatic steatosis and acute liver failure [57]. AFLP typically presents in the second half of pregnancy with significant hepatic dysfunction and characteristic symptoms including nausea, vomiting, abdominal pain, malaise, and anorexia, and may have substantial clinical overlap with HELLP syndrome and severe pre-eclampsia [58,59].

In critically ill patients, findings such as thrombocytopenia, encephalopathy, renal dysfunction, elevated aminotransferases, and multiorgan failure may overlap with TTP. However, hypoglycemia, hyperammonemia, marked coagulopathy due to liver failure, and rapid improvement after delivery are characteristic of AFLP, whereas severe ADAMTS13 deficiency with prominent microangiopathic hemolytic anemia is characteristic of TTP (Table 1) [6,11,59].

### 6.4. Other Conditions

Disseminated intravascular coagulation (DIC) and sepsis-associated thrombocytopenia may coexist and can show partial overlap with TTP in critically ill pregnant patients; however, diagnostic confusion is usually limited because these conditions occur in the setting of an identifiable precipitating event (e.g., sepsis, placental abruption, or postpartum hemorrhage) and are characterized by consumptive coagulopathy [6,51]. Immune thrombocytopenia (ITP) is a common cause of thrombocytopenia in pregnancy but rarely overlaps diagnostically with TTP, as it presents with isolated thrombocytopenia without microangiopathic hemolysis or end-organ dysfunction [60,61]. Drug-induced TMA is a rare differential diagnosis, typically distinguished by a clear temporal association with medication exposure and preserved ADAMTS13 activity [62].

## 7. Diagnostic Work-Up and Risk Stratification in Pregnancy-Associated TTP

**Figure 3:**
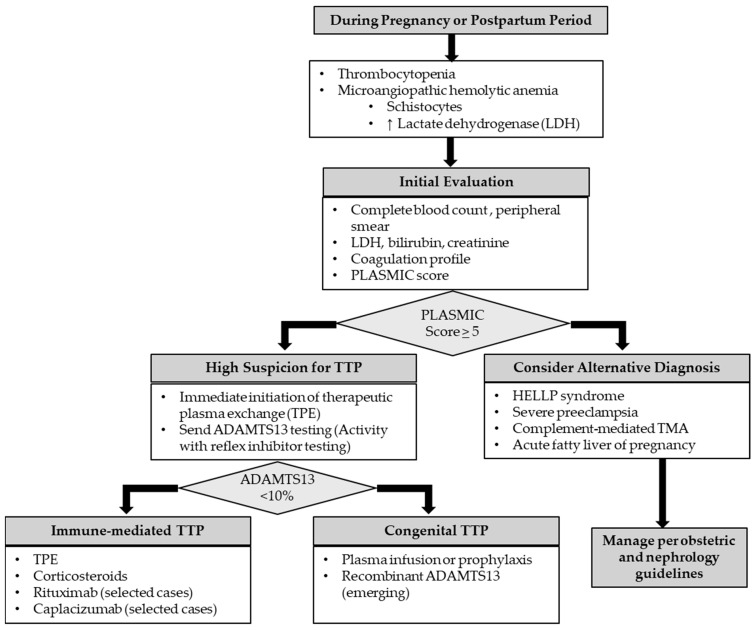
Diagnostic and management algorithm for pregnancy-associated thrombotic thrombocytopenic purpura. The upward arrow means “Increased”.

The diagnosis of thrombotic thrombocytopenic purpura (TTP) relies on careful clinical assessment together with laboratory confirmation of severely reduced ADAMTS13 activity (<10%) [63]. TTP should be strongly suspected in patients presenting with microangiopathic hemolytic anemia (MAHA) and severe thrombocytopenia (platelet count typically < 30,000/µL), with or without evidence of end-organ involvement [23]. As in non-pregnant patients, prompt clinical recognition is critical, as ADAMTS13 activity results are frequently not immediately available and treatment decisions must often be made based on clinical suspicion alone. Accordingly, the standard approach is to establish a presumptive diagnosis of immune-mediated TTP using clinical features and initial laboratory data, as incorporated into validated tools such as the PLASMIC score, and to initiate immediate therapeutic plasma exchange (TPE) without delay (Figure 3) [64,65].

Pregnant or postpartum patients presenting with thrombocytopenia and microangiopathic hemolytic anemia should undergo prompt evaluation, including PLASMIC score assessment and early initiation of therapeutic plasma exchange when clinical suspicion for TTP is high. ADAMTS13 testing facilitates differentiation between immune-mediated and congenital TTP and guides gestation-specific management. Alternative pregnancy-associated thrombotic microangiopathies should be considered when ADAMTS13 activity is not severely reduced [63,64,65,66].

### 7.1. Initial Work Up

When TTP is suspected, a comprehensive initial workup should be initiated, including complete blood count (CBC), peripheral blood smear review, lactate dehydrogenase (LDH), bilirubin, haptoglobin and creatinine, and coagulation profile.

### 7.2. Plasmic Score

The PLASMIC score is a validated clinical prediction tool used to estimate the likelihood of severe ADAMTS13 deficiency. Because ADAMTS13 activity results are frequently not immediately available, clinicians commonly rely on the PLASMIC score in patients with suspected iTTP [63].

The PLASMIC score assigns one point for each of the following variables:

P—Platelet count < 30 × 10^9^/L
L—Hemolysis present (↑ LDH, low haptoglobin, schistocytes, or indirect hyperbilirubinemia)
A—Absence of active cancer
S—Absence of solid organ or stem cell transplant
M—MCV < 90 fL
I—INR < 1.5
C—Creatinine < 2.0 mg/dL

The total score ranges from 0 to 7, with a score of 6–7 strongly supportive of a diagnosis of iTTP, warranting urgent initiation of TPE, including in pregnant patients [63]. A score of 5 indicates intermediate risk, while scores of 0–4 are associated with a low likelihood of TTP. A systematic review and meta-analysis done in 2020 demonstrated that a PLASMIC score ≥ 5 provides very high sensitivity (99%) for severe ADAMTS13 deficiency, while a threshold of ≥6 improves specificity (89%) at the cost of reduced sensitivity (85%) [64]. Given the high maternal and fetal morbidity of untreated TTP and the clinical overlap with other pregnancy-associated TMAs, empiric treatment with TPE in patients with PLASMIC score ≥ 5 is crucial in the absence of a clear alternative diagnosis [11,65].

The PLASMIC score is primarily validated in iTTP rather than congenital TTP and may be useful in differentiating TTP from other pregnancy-associated thrombotic microangiopathies such as HELLP syndrome, acute fatty liver of pregnancy, and DIC. Although the score demonstrates high sensitivity for iTTP, its specificity may be reduced in pregnancy, as physiological changes affecting creatinine and coagulation parameters can influence score components; therefore, clinical judgment remains essential when applying the PLASMIC score in pregnant patients [11,63,66,67].

### 7.3. ADAMTS13 Testing

ADAMTS13 testing (measurement of ADAMTS13 activity with reflex inhibitor testing) is central to the diagnosis and management of TTP. In patients, including pregnant individuals, presenting with MAHA and thrombocytopenia, a severe ADAMTS13 deficiency (<10%) strongly supports the diagnosis of TTP. However, ADAMTS13 activity level >10% does not exclude TTP, particularly when the clinical presentation is highly suggestive and testing is performed after therapeutic intervention (transfusion or TPE) [54]. Importantly, severe ADAMTS13 deficiency (even <10%) has been reported in other causes of MAHA and thrombocytopenia, including sepsis-associated coagulopathy and infection-related secondary TMA, reflecting complex interactions between inflammation, endothelial injury, and ADAMTS13 regulation [68]. Thus, ADAMTS13 results must always be interpreted in the appropriate clinical context.

#### 7.3.1. ADAMTS13 Activity Testing

ADAMTS13 activity is measured using functional assays that report enzyme activity as a percentage of normal pooled plasma. Testing can be performed on plasma or serum, although plasma is preferred. Specimens should be collected prior to plasma transfusion or initiation of TPE, as transfused ADAMTS13 may falsely elevate measured activity into the 11–20% range, potentially obscuring the diagnosis [54].

In rare cases, patients with clinically confirmed TTP may demonstrate ADAMTS13 activity > 20%, which may be attributable to assay variability, inhibitory interference, or timing of sample collection [25,68]. Following recovery from an acute episode of iTTP, some patients may have persistently low ADAMTS13 activity, often with detectable inhibitors, for prolonged periods while remaining clinically asymptomatic [69].

#### 7.3.2. ADAMTS13 Inhibitor Testing

In iTTP, autoantibodies (also called inhibitors) against ADAMTS13 are responsible for severe enzyme deficiency. Inhibitor testing is most commonly performed using mixing studies, in which patient plasma is combined with normal plasma; inhibition of ADAMTS13 activity in the mixture indicates the presence of an inhibitor and allows for titer estimation [70]. Most laboratories reflexively perform inhibitor testing when severe ADAMTS13 deficiency (<10%) is identified.

It is important to note that inhibitor strength does not correlate with disease severity, relapse risk, or survival [71]. In patients with severe ADAMTS13 deficiency without detectable inhibitors, particularly those with childhood onset, recurrent episodes, or presentation during a first pregnancy, cTTP should be strongly considered. Rarely, inhibitors may not be detectable in patients with iTTP due to assay limitations or transient antibody characteristics.

In many institutions, ADAMTS13 testing remains a send-out assay with prolonged turnaround times, which can complicate timely diagnosis and management. Improved accessibility and faster reporting of ADAMTS13 results would significantly enhance care, particularly in pregnancy-associated TMA, where diagnostic overlaps are common [55].

### 7.4. Other Investigations

#### 7.4.1. Genetic Testing

Genetic testing for *ADAMTS13* pathogenic variants should be considered in selected patients to confirm a diagnosis of cTTP, particularly in individuals with severe ADAMTS13 deficiency (<10%) without detectable ADAMTS13 inhibitors. Identification of biallelic *ADAMTS13* variants confirms cTTP and has important implications for long-term management, pregnancy planning, and family counseling [72].

#### 7.4.2. Neuroimaging

Brain imaging with magnetic resonance imaging (MRI) or computerized tomography (CT) should be performed in patients with neurologic symptoms, including headache, altered mental status, seizures, or focal deficits, to evaluate for ischemic or hemorrhagic complications and to exclude alternative diagnoses. Imaging findings may be normal despite significant neurological involvement in TTP [73].

#### 7.4.3. Renal Imaging

Renal involvement in TTP is variable. While kidney dysfunction is often mild, a subset of patients may develop acute kidney injury requiring dialysis, and some may subsequently progress to chronic kidney disease [74]. When renal manifestations are disproportionate, severe, or atypical for TTP, renal ultrasound or other appropriate imaging may be considered to evaluate for structural abnormalities and alternative causes of kidney dysfunction [75].

#### 7.4.4. Fetal Well-Being Assessment

Pregnancy-associated TTP is primarily an acute maternal process, and fetal growth restriction (FGR) is not common compared with other placental disorders such as preeclampsia. Nevertheless, assessment of fetal status remains important to guide the timing of delivery and multidisciplinary management. Obstetric ultrasound, including evaluation of fetal growth, amniotic fluid volume, and fetal surveillance with biophysical profile and Doppler studies, should be performed to assess fetal well-being in high-risk pregnancies [76,77].

## 8. Management of iTTP

### 8.1. Therapeutic Decision-Making Across Gestation in Pregnancy-Associated TTP

#### 8.1.1. Therapeutic Plasma Exchange (TPE)

The mainstay of treatment for iTTP during pregnancy at acute presentation includes urgent TPE in combination with corticosteroids. TPE dramatically reduces mortality in iTTP and should be initiated as soon as the diagnosis is suspected, without waiting for confirmatory ADAMTS13 testing. TPE is performed with PLASMA as the replacement fluid. It acts by removing the circulating anti-ADAMTS13 autoantibodies and ultra-large vWF multimers while at the same time providing ADAMTS13, which is deficient.

The intensity and duration of TPEs during pregnancy are guided by clinical status, platelet count, and markers of hemolysis, particularly LDH levels [78]. TPE is continued until sustained clinical remission, typically defined as normalization of platelet count with improving hemolysis markers, and is maintained for at least 48 h after platelet recovery. Maternal hypotension is a recognized complication of TPE which may lead to transient fetal distress; this is typically managed with volume resuscitation using intravenous crystalloid solutions and hemodynamic support (Table 2) [11,14,53,79,80].

#### 8.1.2. High-Dose Steroids

Glucocorticoids are commonly used in the management of iTTP and are generally considered safe during pregnancy and lactation. Corticosteroids are administered concurrently to suppress autoantibody production. The choice of agent depends on the clinical indication and whether maternal or fetal effects are desired. Dexamethasone is preferred when fetal benefit is intended, such as for the promotion of fetal lung maturation, due to its greater placental transfer. Transfer of glucocorticoids into breast milk is minimal, and neonatal exposure can be further reduced by avoiding breastfeeding for the first 4 h after maternal dosing (Table 2) [81].

#### 8.1.3. Rituximab

Rituximab is a chimeric IgG anti-CD20 monoclonal antibody that suppresses autoantibody production through B-cell depletion. As an IgG antibody, rituximab crosses the placenta, particularly during the second and third trimesters, and has been associated with transient neonatal B-cell lymphopenia, which typically resolves without long-term sequelae [82]. During pregnancy, rituximab is reserved for severe or refractory cases of iTTP that fail to respond to TPE and corticosteroids, and its use should be individualized through multidisciplinary discussion weighing maternal benefit against potential fetal risk (Table 2) [11].

The risk of iTTP relapse is significantly increased in patients with ADAMTS13 activity <10% during remission. In this high-risk population, prophylactic rituximab administered during remission has been shown to reduce relapse rates and restore ADAMTS13 activity [83]. Prophylactic rituximab may be considered in women with persistent severe ADAMTS13 deficiency who are planning pregnancy, and conception is generally deferred for 6–12 months after rituximab exposure to minimize fetal exposure and allow immune reconstitution (Table 2) [84,85].

#### 8.1.4. Caplacizumab

Caplacizumab is a humanized bivalent single-domain (nanobody) monoclonal antibody that binds to the A1 domain of von Willebrand factor (vWF), thereby inhibiting the interaction between ultra-large vWF multimers and the platelet glycoprotein Ib (GPIb) receptor. This mechanism rapidly prevents platelet adhesion and microvascular thrombus formation, which is central to the pathophysiology of iTTP. caplacizumab does not target the underlying autoimmune process and therefore does not suppress anti-ADAMTS13 autoantibody production. As a result, it should be used in conjunction with TPE and immunosuppressive therapy (corticosteroids ± rituximab) and is typically initiated at diagnosis and before or with the first plasma exchange (Table 2) [86].

Clinical trials and real-world studies in non-pregnant iTTP patients have demonstrated that caplacizumab is associated with faster platelet count recovery, reduced exacerbations and refractoriness, and improved short-term outcomes, including reduced disease-related complications [87]. However, pregnancy-specific safety and efficacy data remain scarce, consisting largely of case reports and small case series, with no randomized trials. Thus, use in pregnancy is off-label and should be individualized, generally reserved for selected severe or refractory cases in which anticipated maternal benefit outweighs uncertain fetal risk. Given its antithrombotic mechanism, bleeding is the principal adverse effect and use during pregnancy should be undertaken with caution and within a multidisciplinary framework with careful bleeding risk assessment and shared decision making [88].

#### 8.1.5. Transfusion Principles

In TTP, thrombocytopenia reflects platelet consumption in microvascular thrombi. Therefore, platelet transfusions can potentially worsen thrombosis, leading to adverse outcomes. Platelets may be administered for clinically significant hemorrhage or urgently required cesarean delivery, but such transfusions should be coordinated with TPE to minimize risk [89].

#### 8.1.6. Monitoring Response and Supportive Care

Close and continuous monitoring of both maternal and fetal status is essential throughout pregnancy. Maternal monitoring includes frequent clinical assessment and serial measurement of ADAMTS13 activity, while fetal surveillance should include obstetric ultra-sound with Doppler studies as indicated.

Vaginal delivery is generally preferred when pregnancy progresses without complications and ADAMTS13 activity remains ≥20–25%, corresponding to physiological levels during pregnancy. If ADAMTS13 activity declines below 20–25% in the absence of laboratory or clinical evidence of acute TTP, low-dose corticosteroids may be initiated. If corticosteroids are ineffective or ADAMTS13 activity falls below 10%, prophylactic TPE is initiated, typically on a weekly basis and adjusted according to clinical and laboratory response to prevent relapse [5].

#### 8.1.7. Antithrombotic Therapy

Low-dose aspirin and/or low-molecular-weight heparin (LMWH) may be considered on an individual basis in women with platelet counts >50,000/μL, considering obstetric indications and overall thrombotic risk. However, aspirin is not routinely recommended in iTTP and should be reserved for specific clinical indications rather than used empirically [6].

#### 8.1.8. Refractory/Relapsing iTTP

Refractory TTP is defined as failure to achieve an adequate clinical response or clinical deterioration despite standard therapy, TPE and corticosteroids [90]. Management options for refractory or relapsing iTTP during pregnancy are limited and may include rituximab, caplacizumab, or calcineurin inhibitors such as cyclosporine. These therapies are generally reserved for severe or treatment-resistant disease and should be implemented within a multidisciplinary framework, balancing maternal benefit against potential fetal risk [91].

Pregnant women with a history of iTTP are at increased risk of disease relapse, fetal loss, and hypertensive disorders of pregnancy, including preeclampsia, as pregnancy itself is a well-recognized trigger for relapse. These complications occur more frequently in women with iTTP than in the general obstetric population. ADAMTS13 activity at the start of pregnancy is a strong predictor of relapse risk, and close follow-up with regular interval monitoring of ADAMTS13 activity is essential. In selected high-risk patients, elective or preemptive TPE in the setting of ADAMTS13 activity <10–20% has been associated with improved maternal and fetal outcomes [48].

### 8.2. Management of cTTP

#### 8.2.1. Counseling, Education, and Monitoring

There are important differences in the management of cTTP compared with iTTP. Preconception counseling and detailed risk discussion are essential for women with a known diagnosis of cTTP, as pregnancy is associated with a high risk of disease exacerbation or relapse, which can lead to severe maternal and fetal complications. Close clinical and laboratory monitoring is required throughout pregnancy. Physicians should also provide patients with access to educational materials, online resources, and patient support groups to facilitate understanding and shared decision making [92].

Patients should be educated to recognize early warning signs of relapse and the importance of regular laboratory monitoring, including complete blood counts and ADAMTS13 activity measurements. Blood counts are typically monitored monthly, while ADAMTS13 activity is assessed monthly or every 2–3 months, depending on disease severity and prior course [92,93,94].

#### 8.2.2. Plasma Infusions

Following conception, patients with cTTP should receive prophylactic plasma infusions to prevent disease exacerbation. Although no consensus guidelines exist regarding optimal dosing or frequency, most published studies report that plasma infusion therapy is associated with favorable maternal and fetal outcomes [50]. However, plasma infusion therapy can be burdensome, requiring frequent hospital visits, and may be complicated by allergic reactions, volume overload, and reduced quality of life (Table 2) [50,94].

A commonly used regimen initiates plasma infusion at 10–15 mL/kg twice weekly early in pregnancy. With advancing gestational age, particularly during the second and third trimesters, the dose may be increased to 30–40 mL/kg, while infusion frequency is typically reduced to once or twice weekly, based on clinical response and laboratory parameters [95].

In women with a history of cTTP who do not receive prophylactic therapy, the risk of relapse in subsequent pregnancies is reported to be up to fivefold higher than in those with iTTP, with reported stillbirth rates approaching 40%, largely due to fetal growth restriction, spontaneous abortion, and prematurity [96].

#### 8.2.3. Recombinant ADAMTS13

Recombinant ADAMTS13 (rADAMTS13) has recently been approved by the U.S. Food and Drug Administration for the treatment of patients with cTTP. A phase 3 open-label cross-over trial demonstrated near-complete normalization of ADAMTS13 activity, a reduction in relapse events, and a favorable safety profile, supporting its use as a targeted replacement therapy (Table 2) [97].

Lars M. Asmis and colleagues reported the successful use of recombinant ADAMTS13 during the third trimester in a pregnant patient with cTTP who was refractory to plasma therapy and had severe fetal growth restriction. Administration of rADAMTS13 at a dose of 40 IU/kg resulted in a marked increase in platelet count and ADAMTS13 activity, stabilization of fetal growth, and delivery of a small-for-gestational-age but otherwise healthy infant at 37 weeks’ gestation [95].

Compared with plasma therapy, rADAMTS13 provides more consistent maintenance of ADAMTS13 activity, reduces maternal complications and hospital visits, and may improve fetal outcomes. Planned delivery at approximately 37 weeks’ gestation is generally recommended, as the risk of relapse increases late in pregnancy and plasma infusion alone may be insufficient to prevent disease recurrence [2].

### 8.3. Obstetric and Peripartum Management

Untreated thrombotic TTP during pregnancy is associated with placental infarction, fetal demise, and significant maternal morbidity, including end organ damage. The primary therapeutic goal is to maintain ADAMTS13 activity above approximately 20%, which is associated with improved maternal and fetal outcomes [14].

Management of pregnant patients with TTP requires close multidisciplinary collaboration at specialized centers, involving a hematologist, maternal–fetal medicine specialist, obstetrician, and anesthesiologist. The mode of delivery should be guided by obstetric indications. In the absence of obstetric contraindications, planned induction of labor at 36–37 weeks’ gestation with vaginal delivery is generally recommended for both congenital and immune-mediated TTP. For patients in remission with ADAMTS13 activity ≥25 IU/dL, vaginal delivery is considered appropriate [92].

Fetal indications for earlier delivery include fetal distress and intrauterine growth restriction (IUGR), with prognosis largely dependent on gestational age at birth. Thrombosis of decidual arterioles may lead to placental ischemia and infarction, resulting in impaired fetal growth or fetal demise. Accordingly, serial fetal growth assessments and Doppler ultrasound of uterine and fetal arteries are recommended to monitor placental function and fetal wellbeing. Although maternal anti-ADAMTS13 IgG antibodies can cross the placenta, fetal hematologic involvement is rare [96].

Regional anesthesia is generally preferred over general anesthesia during pregnancy, as it avoids airway manipulation and allows maternal participation during delivery. Thrombocytopenia may limit the use of neuraxial techniques; although an absolute platelet threshold has not been definitively established, many studies suggest a practical lower limit of 70,000–80,000/μL, with lower thresholds reported in selected cases without complications. Anesthetic management should be individualized based on platelet count, urgency of delivery, and multidisciplinary input [98].

## 9. Outcomes

TTP is associated with significant maternal and fetal morbidity and mortality. Affected patients are at increased risk of multisystem involvement, including the cardiac, neurologic, renal, and pulmonary systems. Reported complications include major adverse cardiovascular events, acute kidney injury, acute respiratory distress syndrome, DIC, sepsis, and shock. Advanced maternal age further contributes to increased morbidity compared with the general obstetric population. Distinct patterns of organ involvement have been described, with higher rates of neurological complications in cTTP and increased cardiovascular events in iTTP. Adverse fetal outcomes, including fetal growth restriction and fetal loss, are also more frequent [47].

The risk of relapse during pregnancy is closely associated with both ADAMTS13 activity and gestational age. Relapse risk is low when ADAMTS13 activity is normal at the beginning of pregnancy, and regular monitoring enables timely initiation of elective or preemptive therapy [11]. In the absence of treatment, the risk of recurrence approaches 100% in congenital TTP, whereas relapses occur in approximately 50% of patients with immune-mediated TTP. TTP presenting during the second trimester, particularly between 20 and 29 weeks’ gestation, is associated with higher rates of fetal loss and intrauterine growth restriction in both congenital and acquired forms. In contrast, pregnancy outcomes are generally more favorable when disease onset occurs early (<20 weeks) or late (>30 weeks) in gestation [11]. Several studies have reported improved fetal prognosis when TTP is identified and treated during the third trimester, likely reflecting the presence of a fully developed placenta that is better able to tolerate microvascular injury and hemodynamic disturbances. In contrast, disease onset earlier in pregnancy may compromise placental development and function, resulting in adverse fetal outcomes [11,99,100].

Pregnancy outcomes are worse in women experiencing a first episode of TTP during pregnancy compared with those with recurrent disease. Severe ADAMTS13 deficiency (<10%) is strongly associated with disease activity and represents an indication for TPE and immunosuppressive therapy to improve maternal and fetal outcomes [101].

## 10. Special Situations

### 10.1. TTP in the Postpartum Period

TTP may occur during the postpartum period, with several cases reported in the literature. Prompt recognition and early initiation of therapy are critical, as delayed treatment is associated with significant maternal mortality [102]. Physiological restoration of maternal immune homeostasis after delivery may precipitate disease onset in susceptible individuals [6]. A high index of suspicion should therefore be maintained in postpartum patients presenting with unexplained thrombocytopenia, hemolytic anemia, or neurologic symptoms [103].

### 10.2. Pregnancy-Associated TTP in Patients with Systemic Lupus Erythematosus (SLE)

TTP is uncommon in patients with SLE, with a reported incidence of approximately 0.5%, although the true prevalence may be underestimated [104]. Pregnancy-associated TTP may be triggered by underlying SLE activity or concurrent infections. Chronic immunosuppressive therapy, particularly corticosteroids, may alter clinical presentation and delay diagnosis during pregnancy. Consequently, TTP should remain an important differential diagnosis in pregnant patients with SLE who develop thrombocytopenia or microangiopathic hemolytic anemia [5].

Diagnosis is often challenging because of overlapping clinical and laboratory features between SLE-associated thrombotic microangiopathy and TTP. Careful review of the peripheral blood smear is essential, as the presence of >1% schistocytes in the absence of alternative causes of thrombocytopenia supports a diagnosis of TTP and should prompt early treatment. These patients are at increased risk of infection, particularly in intensive care unit settings, and preventive strategies should be implemented during treatment. Currently, there are no standardized guidelines for modifying TTP therapy in the setting of severe infection. Overall, TTP associated with SLE is linked to adverse outcomes, higher mortality, and reduced response to therapeutic plasma exchange [104,105].

### 10.3. TTP Associated with Human Immunodeficiency Virus (HIV)

HIV-associated TTP remains more prevalent in developing countries than in developed regions, likely reflecting differences in access to antiretroviral therapy (ART). Pathophysiology is multifactorial and incompletely understood [106]. TTP typically occurs in patients with high viral loads and low CD4 counts, although cases have also been reported in individuals with preserved CD4 levels. Clinical presentation may include severe thrombocytopenia and prominent neurologic manifestations, with relatively mild renal involvement. In addition to plasma exchange therapy, initiation of ART is essential to prevent disease recurrence. Without treatment, HIV-associated TTP carries a high mortality risk [106,107].

### 10.4. Other Viral–Associated and COVID-19–Related TTP

Secondary TTP has been reported in association with both DNA and RNA viruses. DNA viruses, such as cytomegalovirus (CMV) and human herpesvirus 8 (HHV-8), and RNA viruses, including human T-cell lymphotropic virus (HTLV) and influenza, may induce TTP through direct endothelial injury and inhibition of ADAMTS13 activity [108].

Multiple mechanisms have been proposed for COVID-19-associated TTP, including endothelial cytopathic injury, elevated levels of von Willebrand factor, factor VIII, fibrinogen, and D-dimer, as well as reduced ADAMTS13 activity driven by systemic inflammation. Clinical presentation may be atypical and not always fulfill classic TTP criteria. Except for two studies, most reports have found no clear correlation between COVID-19 disease severity and ADAMTS13 activity [109]. Management includes therapeutic plasma exchange and corticosteroids, along with treatment of the underlying infection. The use of rituximab should be approached cautiously, as it may predispose to viral reactivation or secondary infection [110].

## 11. Long-Term Follow-Up

The risk of relapse remains a major concern in patients with TTP, including pregnancy-associated TTP, both during the postpartum period and in subsequent pregnancies. Serial monitoring of ADAMTS13 activity is therefore essential to stratify relapse risk and to guide preemptive therapy. After achieving remission, ADAMTS13 activity should be monitored monthly for the first 3 months, followed by quarterly assessments up to 1 year. If ADAMTS13 activity remains stable and within the normal range, monitoring frequency may subsequently be reduced to once or twice annually. A decline in ADAMTS13 activity to <10% is strongly predictive of relapse, and preemptive treatment with rituximab has been shown to reduce recurrence risk in immune-mediated TTP (iTTP) [111].

In iTTP, long-term follow-up should focus on early detection of recurrent immune-mediated ADAMTS13 deficiency, particularly in women planning future pregnancies. Patients with cTTP are at near universal risk of recurrence without prophylactic therapy. Lifelong ADAMTS13 deficiency in cTTP necessitates regular prophylactic plasma infusion or recombinant ADAMTS13 replacement, with intensified surveillance and prophylaxis during pregnancy and the postpartum period.

Pregnancy-associated TTP is a life-altering condition with long-lasting effects that extend beyond the acute episode. Survivors frequently experience persistent fatigue, anxiety, depression, cognitive impairment, and behavioral changes, which may be exacerbated during pregnancy and the postpartum period. These symptoms can significantly impair occupational functioning and reduce engagement in social and family activities [112]. Clinicians should remain vigilant for acute neurological complications, including stroke and transient ischemic attacks, as well as long-term neuropsychiatric sequelae such as anxiety, depression, and cognitive dysfunction, all of which substantially impact quality of life [113].

Given the complex medical, psychological, and reproductive implications of pregnancy-associated TTP, a multidisciplinary care model involving hematology, maternal–fetal medicine, neurology, and mental health professionals is essential to optimize long-term maternal outcomes and to support safe future pregnancies [112].

## 12. Future Direction

TTP, although rare, is a life-threatening emergency in which delays in treatment markedly increase morbidity and mortality. A major barrier to timely diagnosis is that ADAMTS13 activity testing often involves a send-out assay, leading to prolonged turnaround times and empiric exposure to TPE and immunosuppression in patients who ultimately do not have iTTP or even have cTTP.

Rapid ADAMTS13 activity assays have therefore emerged as a key advance in accelerating diagnostic certainty and improving early triage. The automated chemiluminescent quantitative ADAMTS13 activity assay can provide results in <1 h and has shown good concordance with conventional ELISA methods in patients with high clinical suspicion of TTP. This assay uses magnetic particles coated with a von Willebrand factor (vWF73) substrate. Cleavage of vWF73 by ADAMTS13 is detected using an isoluminol-labeled monoclonal antibody, yielding a chemiluminescent signal proportional to enzymatic activity. However, when pretest probability is low (in conditions like sepsis- or malignancy-associated TMA), interpretation requires caution because low ADAMTS13 activity can occur in critical illness and may lead to misleading results if used without an appropriate diagnostic algorithm [114]. More broadly, a recent systematic review/metanalysis supports that rapid ADAMTS13 assays can aid diagnostic accuracy and reduce unnecessary therapies when integrated into structured pathways [115].

Growing evidence supports the role of ADAMTS13 biomarkers in predicting disease relapse and guiding long-term management. Consensus data indicate that ADAMTS13 activity levels below 10% during clinical remission are strongly associated with an increased risk of subsequent relapse. In addition to functional activity, several studies have highlighted the prognostic value of ADAMTS13 antigen levels, with lower antigen concentrations correlating with more severe disease, poorer outcomes, and higher rates of recurrence and exacerbation. ELISA-based assays are more sensitive than functional assays for detecting anti-ADAMTS13 autoantibodies, and the presence of anti-ADAMTS13 IgG has been associated with an increased risk of disease exacerbation following therapy [116].

Recent advances have further characterized the structural biology of ADAMTS13, which exists in either a closed (inactive) or open (active) conformation. The closed conformation predominates in healthy individuals, whereas the open conformation reflects the presence and pathogenic activity of anti-ADAMTS13 autoantibodies. Notably, ADAMTS13 autoantibodies may persist in some patients in clinical remission despite ADAMTS13 activity levels exceeding 50%. In such individuals, an elevated open conformation index (>0.645) has been shown to predict earlier relapse, suggesting a potential role for conformational analysis in refined risk stratification and personalized disease monitoring [117].

Rapid ADAMTS13 activity testing should be viewed not merely as a diagnostic assay but as an integral component of a care pathway. The future of TTP management lies in the integration of rapid ADAMTS13 testing with PLASMIC score-based algorithmic approaches, enabling timely diagnosis while minimizing unnecessary exposure to TPE and caplacizumab in patients with non-TTP thrombotic microangiopathies [115]. Emerging data also suggest that rapid assays may be cost-effective compared with traditional send-out testing, particularly in settings where empiric caplacizumab is incorporated into initial management strategies [118].

Beyond acute diagnosis, relapse risk signals may persist even when ADAMTS13 activity appears normalized, and assessment of ADAMTS13 open–closed conformational dynamics may complement conventional monitoring to refine relapse prediction and personalize long-term management [117,119]. Finally, variability in anti-ADAMTS13 antibody assays across platforms underscores the need for standardized testing approaches and improved antibody profiling, representing an important area for future laboratory development and harmonization [120].

## 13. Conclusions

Pregnancy-associated TTP remains a rare but critical TMA in which delayed recognition can rapidly translate into preventable maternal and fetal morbidity. The central diagnostic challenge is its substantial clinical and laboratory overlap with preeclampsia/HELLP, CM- TMA, AFLP, and sepsis-associated thrombocytopenia. Across gestation, the most reliable distinguishing marker remains severe ADAMTS13 deficiency (<10%), and management hinges on acting on clinical suspicion rather than waiting for confirmatory testing. In iTTP, urgent TPE plus corticosteroids remain the cornerstone, with escalation to rituximab in refractory or severe cases. Although caplacizumab is a promising adjunct in severe or refractory iTTP, its use in pregnancy remains off-label and it should be reserved for selected high-risk situations with careful bleeding-risk assessment and shared decision-making. In cTTP, anticipatory diagnosis, genetic confirmation when indicated, and prophylactic plasma or targeted replacement with rADAMTS13 play key roles in optimizing outcomes. Multidisciplinary care and structured monitoring, particularly serial ADAMTS13 activity, are essential to improve peripartum safety, mitigate relapse risk, and guide future pregnancy planning. Looking ahead, broader access to rapid ADAMTS13 assays, and pregnancy-specific pathways that integrate clinical risk tools with biomarker-driven decisions may reduce diagnostic uncertainty, minimize unnecessary exposure to TPE, and improve precision management for this uniquely complex obstetric emergency.

## Figures and Tables

**Table 1:** Key clinical and laboratory features differentiating pregnancy-associated thrombotic microangiopathies.

Feature	TTP	HELLP Syndrome	C-TMA/aHUS	AFLP
Typical Timing	Any trimester but often in 2nd to 3rd trimester	Third trimester or immediate postpartum	Predominantly postpartum	Late third trimester and may also in the early postpartum period
ADAMTS13 activity	Severely reduced (<10%)	Normal or mildly reduced	Normal or mildly reduced	Normal
Neurologic manifestations	Transient focal neurologic deficits (confusion, aphasia, weakness, seizures)	Severe headache, visual disturbances, hyperreflexia	Less common	Encephalopathy
Renal involvement	Mild or absent	Mild–moderate AKI	Severe AKI common, often dialysis-requiring	Mild–moderate AKI, sometimes severe
Platelet count	Severe thrombocytopenia (often <20 × 10^9^/L) that persists after delivery	Thrombocytopenia that typically improves after delivery	Mild–moderate thrombocytopenia	Mild–moderately decreased
Hemolysis (MAHA)	Marked	Present	Present	Absent or mild if present.
Lactate dehydrogenase/Aspartate Aminotransferase Ratio	Elevated LDH/AST ratio (>22), reflecting MAHA	Lower LDH/AST ratio (<22)	Elevated LDH/AST ratio (LDH elevated; AST usually mild)	Lower LDH/AST ratio (more primary hepatic failure)
Response to Delivery	No improvement	Rapid improvement	Often worsens postpartum	Typically improved after delivery

Note. TTP, Thrombotic Thrombocytopenic Purpura; HELLP, Hemolysis, Elevated Liver enzymes, Low Platelet count; C-TMA, Complement-Mediated TMA; aHUS, Atypical hemolytic uremic syndrome; AFLP, Acute Fatty Liver of Pregnancy; MAHA, Microangiopathic hemolytic anemia; AKI, Acute kidney injury.

**Table 2:** Pregnancy-specific treatment strategies for Immune-mediated TTP vs. Congenital TTP.

Therapy	iTTP	cTTP	Pregnancy Considerations
Therapeutic plasma exchange (TPE)	First line treatment. Must be initiated immediately initiated	No	Generally safe in pregnancy
Corticosteroids	Adjunct to TPE	No	Generally considered safe
Rituximab	Refractory disease or preemptive therapy (e.g., rising inhibitor/declining ADAMTS13 in known iTTP)	No	Used case-by-case; often avoided in 1st trimester; may be considered in 2nd–3rd trimester when maternal benefit outweighs risks
Caplacizumab	High-risk, refractory, or severe iTTP; used with TPE + immunosuppression	No	Limited pregnancy data; bleeding risk, multidisciplinary decision with obstetrics/anesthesia
Plasma infusions	Not standard (may be temporizing only if TPE unavailable)	Cornerstone therapy (Episodic or prophylactic to maintain ADAMTS13 activity)	Safe; used prophylactically during pregnancy to prevent relapse
Recombinant ADAMTS13	No	Emerging disease-specific therapy (may reduce need for plasma)	Pregnancy data limited; theoretical benefit in cTTP; use in specialized centers or clinical trials

Note. iTTP, immune-mediated thrombotic thrombocytopenic purpura; cTTP, congenital thrombotic thrombocytopenic purpura; TPE, therapeutic plasma exchange.

## Data Availability

All relevant data are included in the manuscript.

## Abbreviations

The following abbreviations are used in this manuscript:

ADAMTS13	A Disintegrin and Metalloprotease with ThromboSpondin type 1 motif, member 13
aHUS	Atypical hemolytic uremic syndrome
AFLP	Acute fatty liver of pregnancy
ART	Antiretroviral therapy
CBC	Complete blood count
CM-TMA	Complement-mediated thrombotic microangiopathy
CT	Computed tomography
cTTP	Congenital thrombotic thrombocytopenic purpura
DIC	Disseminated intravascular coagulation
FGR	Fetal growth restriction
HELLP	Hemolysis, Elevated Liver enzymes, and Low Platelet count
HIV	Human immunodeficiency virus
HUS	Hemolytic uremic syndrome
ICU	Intensive care unit
INR	International normalized ratio
iTTP	Immune-mediated thrombotic thrombocytopenic purpura
ITP	Immune thrombocytopenia
LDH	Lactate dehydrogenase
LMWH	Low-molecular-weight heparin
MAHA	Microangiopathic hemolytic anemia
MCV	Mean corpuscular volume
MRI	Magnetic resonance imaging
PT	Prothrombin time
sC5b-9	Soluble terminal complement complex
SLE	Systemic lupus erythematosus
TMA	Thrombotic microangiopathy
TPE	Therapeutic plasma exchange
TTP	Thrombotic thrombocytopenic purpura
UL-vWF	Ultra-large von Willebrand factor multimers
vWF	von Willebrand factor
